# Bulevirtide in Chronic Hepatitis D Patients Awaiting Liver Transplantation Results From a French Multicentric Retrospective Study

**DOI:** 10.1111/liv.70033

**Published:** 2025-02-17

**Authors:** Magdalena Meszaros, Marie‐Noëlle Hilleret, Jérôme Dumortier, Louis D'Alteroche, Armand Abergel, Marianne Latournerie, Teresa Antonini, Filomena Conti, Patrick Borentain, Sébastien Dharancy, Georges‐Philippe Pageaux

**Affiliations:** ^1^ Liver Transplantation and Hepatogastroenterology Unit CHU Montpellier, CHU Montpellier Montpellier France; ^2^ Hepato‐Gastroenterology Unit CHU Grenoble Alpes Grenoble France; ^3^ Digestive Diseases Federation Edouard Herriot Hospital, Hospices Civils de Lyon, Université Claude Bernard Lyon 1 Lyon France; ^4^ Hepatogastroenterology Unit CHU Tours Tours France; ^5^ Hepatogastroenterology Unit CHU Estaing Clermont‐Ferrand Clermont‐Ferrand France; ^6^ Hepatogastroenterology Unit CHU Dijon Dijon France; ^7^ Hepatology Unit Hôpital de la Croix‐Rousse Lyon France; ^8^ Hepatology Unit Pitié Salpêtrière, Assistance Publique‐Hôpitaux de Paris Paris France; ^9^ Hepatogastroenterology Unit CHU Timone Marseille France; ^10^ Hepatology Unit Claude Huriez Hospital, CHU Lille Lille France; ^11^ University of Montpellier Montpellier France

**Keywords:** bulevirtide, chronic hepatitis D, liver transplantation, safety, virological response

## Abstract

**Background and Aims:**

The impact of bulevirtide in patients awaiting liver transplantation (LT) for decompensated liver disease and/or hepatocellular carcinoma (HCC) is unclear. We assessed clinical, virological, and biochemical responses to bulevirtide in patients with chronic hepatitis delta virus (HDV) awaiting LT and compared outcomes with a cohort of similar untreated patients.

**Methods:**

Consecutive HDV‐infected patients waiting for LT since bulevirtide approval were included. Patients receiving 2 mg of bulevirtide daily had clinical, biological, and virological data collected at baseline, Week 24, Week 48, at LT, and post‐LT. Patients not receiving bulevirtide had data collected at baseline, LT, and post‐LT for comparison.

**Results:**

Forty‐one patients from nine LT centers were included. In the bulevirtide group (20 patients; mean age 52.8 ± 9.98 years; 75% male), 65%, 10% and 25% were Child‐Pugh A, B and C, respectively. Fifteen completed 48 weeks of therapy. At 48 weeks, median HDV RNA decreased by 2.56 log IU/mL (*p* = 0.004). Virological and biochemical responses were obtained in 73.3% and 66.6% of patients. Twelve patients (60%) underwent LT. No serious adverse events occurred. Bulevirtide improved liver function, enabling one (7.1%) HCC patient to undergo chemoembolization while on the WL and leading to delisting of three (15%) other patients. In untreated patients (mean age 42.9 ± 7.9 years; 76.2% Child‐Pugh C), none were delisted. Three‐month transplant‐free survival was 76.9% in the bulevirtide group versus 36.7% (*p* = 0.007) in the control group.

**Conclusions:**

Bulevirtide demonstrates safety and efficacy in HDV‐infected patients listed on the LT waiting list and may potentially improve pre‐transplant outcomes.

**Abbreviations**
AFP: alpha‐fetoprotein
ALT: alanine aminotransferase
BCLC: Barcelona clinic liver cancer
BLV: bulevirtide
CI: confidence interval
EMA: European Medicines Agency
HBsAg: hepatitis B surface antigen
HBV: hepatitis B virus
HCC: hepatocellular carcinoma
HDV: hepatitis delta virus
IQR: interquartile range
LSM: liver stiffness measurement
LT: liver transplant
MELD: model for end‐stage liver disease
NA: nucleos(t)ide analogs
NTCP: Na^+^‐taurocholate co‐transporting polypeptide
PegIFNα: PEGylated interferon alpha
RNA: ribonucleic acid
UI/mL: international units per millilitre

**Summary**

- Bulevirtide use in patients awaiting for liver transplant for HCC and/or decompensated liver disease was safe and allowed improvement in liver function in some particular cases.
- At week 48 of treatment, 73.3% of patients had virological response and 66.6% had biochemical response.
- Bulevirtide facilitated HCC treatment in patients on the LT waiting list.
- Three (15%) cirrhotic decompensated patients were delisted due to improvement in liver function while on treatment.
- Post liver transplant outcomes were favourable for a majority of patients.

## 1 Introduction

Chronic hepatitis delta (CHD) poses a significant global health burden as it is the most severe form of chronic viral hepatitis. Patients with CHD are at an increased risk of developing liver cirrhosis, decompensated end‐stage liver disease and hepatocellular carcinoma (HCC) [1, 2, 3, 4]. The exact prevalence of CHD remains uncertain due to limited routine testing, but CHD is thought to affect ~2%–5% of chronic hepatitis B (CHB) carriers [5, 6].

Bulevirtide, a pioneering hepatitis B virus (HBV) entry inhibitor, has emerged as a promising therapeutic agent for CHD. By mimicking the sodium taurocholate co‐transporting polypeptide (NTCP) receptor‐binding domain, bulevirtide disrupts the entry of HDV and HBV into hepatocytes, thereby blocking viral spread [7, 8]. Phase 2 trials have demonstrated encouraging efficacy and safety profiles for bulevirtide, leading to its conditional approval for the treatment of compensated CHD by the European Medicines Agency (EMA) in July 2020 and full marketing authorization on July 2023 [8, 9, 10, 11]. In France, bulevirtide has been available since September 2019 through an early access program. However, the optimal duration of bulevirtide treatment is unknown, and the current guidelines recommend long‐term treatment [10].

Currently, there are no licensed treatments for CHD‐related decompensated liver disease. Liver transplantation (LT) is the best option, with hepatitis B immunoglobulin (HBIg) and nucleos(t)ide analogs (NA) administered as standard prophylaxis post‐transplantation to avoid HBV recurrence [12]. It is unclear whether bulevirtide treatment in patients on the LT waiting list for decompensated liver disease changes the disease course or helps downstage HCC patients as a bridge to LT. Therefore, this study aimed to gather real‐life data on bulevirtide use, safety and efficacy in patients awaiting LT or undergoing evaluation for LT for either decompensated cirrhosis or HCC and compare these data to a cohort of patients not receiving bulevirtide.

## 2 Patients and Methods

### 2.1 Patients

All consecutive HDV‐infected patients with cirrhosis who were on the liver transplant waiting list or underwent pretransplant evaluation since bulevirtide approval, whether prescribed bulevirtide alone or not, were included in this multicenter study in France. The study was conducted between January and October 2024 in accordance with the Declaration of Helsinki. All patients provided written informed consent, and the study was approved by the Institutional Review Board of Montpellier University Hospital (Number 2024‐05‐053). CHD was defined as persistent HDV RNA for more than 6 months. Cirrhosis was defined either noninvasively (liver stiffness measurement > 12 kPa), histologically (F4 stage fibrosis by METAVIR scoring), or by compatible clinical, laboratory and imaging data. Indications for liver transplant evaluation and waiting list inscription were decompensated cirrhosis, defined by overt ascites, encephalopathy or variceal bleeding. The Model for End‐stage Liver Disease (MELD) score was considered for prioritisation, with a minimum score of 15 required for candidacy. LT was also considered in cases of specific exceptions to MELD scores or in cases of unresectable HCC, considering the French alpha‐fetoprotein (AFP) model, provided that the AFP score was ≤ 2 [13, 14, 15].

### 2.2 Treatment

Bulevirtide was self‐administered subcutaneously every 24 h. The decision to start bulevirtide treatment, treatment duration, the decision to co‐prescribe nucleos(t)ide analogs (NA; entecavir or tenofovir) or PegIFNα, and post LT treatment was at the discretion of the investigators.

### 2.3 Follow Up and Data Collection

Clinical, biological and virological characteristics were collected at bulevirtide initiation (baseline), Week 24 (W24), Week 48 (W48), LT and after LT. For patients not receiving bulevirtide, data were collected at baseline (inscription on the LT list), LT, and after LT. Liver‐related events (ascites, variceal bleeding, encephalopathy, liver failure, HCC or LT) and adverse events were documented. Upper endoscopy was performed according to the current guidelines [15]. Imaging for HCC surveillance was performed every 3 months as tumour status, AFP level, and MELD score must be updated at least every 3 months for all patients on the national waiting list for LT. Liver stiffness measurements (LSM) were performed using FibroScan (Echosens, Paris, France) at the discretion of the investigator, by a trained operator according to the manufacturer's recommendations, and following defined quality criteria [16].

Hepatic encephalopathy was graded according to the West Haven criteria [17], and ascites were graded based on the International Ascites Club classification [18].

HDV RNA was quantified using the EurobioPlex assay (Eurobio Scientific, Les Ulis, France), with a lower limit of detection of 20 UI/mL. HBV DNA and HBsAg were quantified using the Abbott Alinity i platform (Abbott Laboratories, Chicago, IL) according to the manufacturer's instructions, with a lower limit of detection of < 10 UI/mL. Retrospective data collection was conducted using medical files and the CRISTAL database, which is maintained by the French Agency for Transplantation (Agence de la Biomedicine) and prospectively records data for all patients on the LTwaiting list.

### 2.4 Outcomes

The primary outcome was virological response at W48, defined as undetectable HDV RNA or ≥ 2‐log decrease compared to baseline in bulevirtide‐treated patients.

Secondary outcomes were as follows: (1) virological non‐response defined as < 1 log HDV RNA decrease versus baseline in bulevirtide‐treated patients; (2) biochemical response defined as ALT normalisation versus baseline in bulevirtide‐treated patients; (3) combined response defined as both a virological and biochemical response in bulevirtide‐treated patients; (4) percentage of patients undergoing LT; (5) percentage of patients with liver‐related events during bulevirtide treatment or while on the waiting list (liver decompensation, new HCC, or HCC progression while on the waiting list); (6) safety of bulevirtide treatment; and (7) comparison between bulevirtide‐treated and untreated groups, including reasons for treatment abstention.

### 2.5 Statistical Analysis

Statistical analyses were performed using EasyMedStat (v3.32; www.easymedstat.com). Continuous variables are expressed as mean (±SD) for normally distributed data and as median and range for non‐normally distributed data. Categorical variables are presented as absolute and relative (%) frequencies. Categorical variables were compared using the chi‐squared or Fisher's exact tests and continuous variables by Student's *t*‐test, Mann–Whitney *U* test, or Kruskal–Wallis test, as appropriate. Repeated analyses were performed using the Friedman test. If the null hypothesis of this test was rejected, *post hoc* pairwise analyses were performed using Nemenyi's test. The Kaplan–Meier method was used to estimate survival probabilities from baseline until LT. The log‐rank non‐parametric test for the comparison of survival distributions was used to compare the survival differences. The alpha risk was set at 5%, and two‐tailed tests were used. The difference between baseline and W48 HDV RNA levels was assessed using the Wilcoxon signed‐rank test.

## 3 Results

### 3.1 Baseline Characteristics

Since the approval of Bulevirtide in France in September 2019, 41 HDV‐infected patients with cirrhosis have been listed for LT across nine French liver transplant centers. The patients were retrospectively included in this study between January and October 2024. The median time between BLV initiation and inscription on the LT was 5.68 (±12.51) months.

Twenty (48.8%) patients received bulevirtide while on the LT waiting list (bulevirtide group). Table 1 presents the baseline patient characteristics. The mean age of treated patients was 52.8 ± 9.98 years, and 75% were male. At baseline, 13 patients (65%) were classified as Child‐Pugh A, two (10%) as Child‐Pugh B, and five (25%) as Child‐Pugh C. Median baseline HDV RNA was 5.98 (2.23–10) log IU/mL. Concomitant PegIFNα was prescribed to three (15%) patients at the discretion of the investigator while on the LT waiting list. None of them presented a prior decompensation episode but all stopped PegIFNα before W24 due to: intolerance (1 patient) or after a decompensation episode (2 patients). For this 2 patients stopping PegIFNα resulted in recompensation of liver function and did not change evolution of liver disease. NAs were prescribed to 90% of patients, and two patients (10%) received bulevirtide monotherapy without NA, at the discretion of the investigators, as they had compensated liver disease and HDV DNA was undetectable.

**TABLE 1:** Baseline characteristics of patients included in the study since BLV approval in 2019 (*n* = 41).

Characteristic	BLV group (*n* = 20)	Control group (*n* = 21)	*p*
Age, mean (SD), years	52.8 (**±**9.98)	42.94 (± 7.9)	0.001
Male sex No. (%)	15 (75)	12 (57.14)	0.32
Liver disease requiring LT (No. %)			
Refractory ascites	1 (5%)	9 (42.9%)	0.001
Active HCC at baseline	8 (40%)	6 (28.5)	0.008
BCLC stage O	4 (50%)	0 (0%)	
BCLC stage A	1 (12.5%)	6 (28.5)	
BCLC stage B	3 (37.5%)	0 (0%)	
Decompensated cirrhosis	7 (35%)	20 (95.23%)	0.001
Compensated cirrhosis at baseline but active HCC on treatment	5 (25%)	1 (4.7%)	
Compensated cirrhosis and inactive HCC at baseline	1 (5%)	0 (0%)	
Child‐Pugh score			
A	13 (65%)	1 (4.76%)	0.001
B	2 (10%)	4 (19.05%)	
C	5 (25%)	16 (76.19%)	
MELD score	9 (6–32)	19.9 (7–40)	0.001
ETV or TDF treatment	18 (90%)	21 (100%)	0.41
LSM median (range)^a^	24.16 (10–50)		
Large oesophageal varices (grade II–III)^b^	11 (57.9%)	10 (47.6%)	0.38
Previous PegIFNα treatment	5 (25%)	3 (14.2%)	0.75
Ongoing PegIFNα treatment^d^	3 (15%)	0 (0%)	
Laboratory tests on baseline^e^			
Albumin, g/L	34.3 (29–39.8)	27 (20–45)	0.001
Platelet count × 10^3^/mmol/L	94 (50.2–285.5)	70.6 (20–179)	0.1
Creatinine mmol/L	69.01 (51.5–84.5)	102.5 (51–480)	0.42
AST, U/L	79 (65.5–125)	74.5 (60–136)	0.75
ALT, U/L	91 (60–135.5)	43.5 (18–353)	0.02
Bilirubin, mmol/L	20.7 (10–444)	56 (18–618)	0.004
Prothrombin time %	78 (26–100)	41.5 (14–62)	0.002
HBsAg, IU/mL^c^	4171 (1620–4908.5)	1930.0 (± 1619.77)	0.1
HBeAg negative	18 (90%)	19 (90%)	0.9
HBV DNA detectable (range 12–4620 UI/mL)	5 (25%)	9 (42.8)	0.38
HDV RNA, log IU/mL	5.98 (2.23–10)	4.61 (± 2.96)	0.38

Abbreviations: ALT, alanine aminotransferase; AST, aspartate aminotransferase; BCLC, barcelona clinic liver cancer; ETV, entecavir; HBV, hepatitis B virus; LSM, liver stiffness measurement; MELD, model for end‐stage liver disease; PegIFNα, PEGylated interferon alpha; TDF, tenofovir disoproxil fumarate.

^a^ *n* = 9 patients.

^b^ *n* = 19 patients.

^c^ *n* = 13 patients.

^d^ *n* = 2 patients received 180 g/week and 130 μg/week.

^e^ Results are presented as median and range (min‐max).

Active HCC was present at baseline in eight patients (40%), with four (50%) classified as BCLC‐0, three (37.5%) as BCLC‐B, and one (12.5%) as BCLC‐A.

Twenty‐one (51.2%) patients did not receive BLV treatment while on the LT waiting list (control group), the characteristics are also summarised in Table 1. The mean age of these patients was 42.94 ± 7.9 years. At baseline, nine patients (42.9%) were classified as having refractory ascites, six patients (28.5%) had active hepatocellular carcinoma (HCC), and 20 (95.23%) patients had decompensated cirrhosis, with 16 (76.19%) classified as Child‐Pugh C.

### 3.2 Virological and Biochemical Responses in Bulevirtide Treated Patients on the Waiting‐List

Median time of BLV treatment was 86.90 (±61.4) weeks and 12 (60%) patients received BLV beyond the Weeks 48 of treatment. Fifteen (75%) patients completed 48 weeks of BLV treatment while on the LT waiting list. The kinetics of HDV RNA is shown in Table 2. Among the five patients who did not complete 48 weeks of treatment, two were transplanted between Week 24 and 48 for compensated cirrhosis and active HCC, one was transplanted earlier for decompensated cirrhosis after having less than 24 weeks of treatment, and two patients were still on treatment but had less than 48 weeks of treatment at the end of the study.

**TABLE 2:** Progression of viral parameters for included patients receiving bulevirtide (*n* = 20 patients).

	Baseline *N* = 20	Week 24 *N* = 17	Week 48 *N* = 15	At LT *N* = 12
HDV RNA, log IU/mL	5.98 (2.23–10)	4.53 (0.15–10)	2 (0.1–7.5)	3.11 (0.1–6.37)
HDV RNA decrease > −2 log IU/mL vs. baseline	—	7 (41.18%)	11 (73.33%)	4 (33.33%)
HDV RNA undetectable	0	3 (18.7%)	8 (53.33%)	3 (37.5%)
HDV RNA decrease < 1 log UI/mL vs. baseline	—	4 (23.53%)	3 (20%)	4 (33.33%)
HBV DNA undetectable	15 (75%)	14 (82.35%)	14 (93.3%)	11 (91.6%)
HBs Ag UI/mL	4171 (1620–4908.5)^a^	4700 (2519–8580)^a^	2629 (111–12854.81)^b^	2186 (200–5976)^b^
Virological response		7 (41.18%)	11 (73.33%)	4 (33.33%)

*Note:* Results are presented as median and range (min‐max).

^a^ *n* = 11 patients.

^b^ *n* = 10 patients.

**FIGURE 1:**
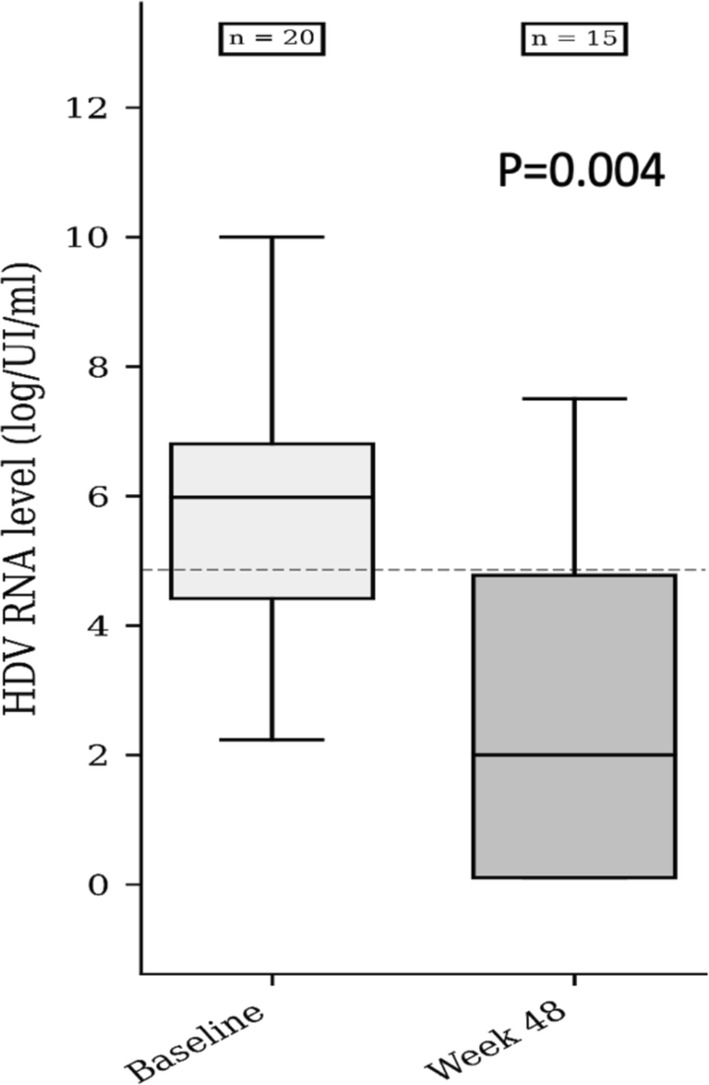
HDV RNA levels from baseline to week 48.

**FIGURE 2:**
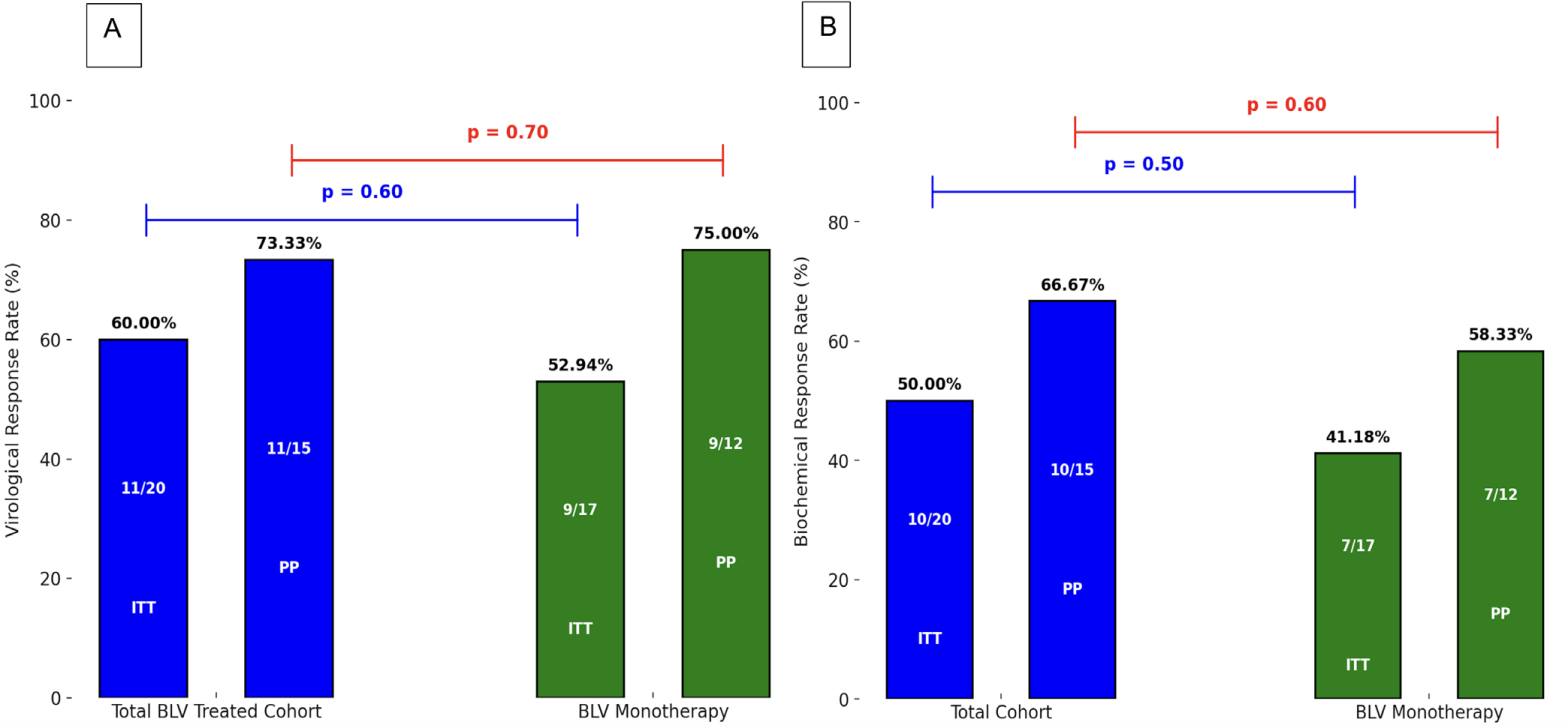
(A)virological and (B) biochemical response rates in bulevirtide‐treated cohort (total cohort) and Bulevirtide (BLV) monotherapy groups: Per‐protocol (PP) and intention‐to‐treat (ITT) analyses.

In per‐protocol analysis (PP) after excluding these (*n* = 5) patients the median HDV RNA level at Week 48 was 2.0 log IU/mL (IQR 4.68). The median difference in HDV RNA between baseline and W48 was 2.56 log IU/mL IQR = 3.69; 95% CI 0.97–4.91; *p* = 0.004; (Figure 1). At W48, HDV RNA was undetectable in eight (53.33%) patients. Virological response at W48 occurred in 11 (73.3%) patients and non‐response in three (20%) patients. In intention‐to‐treat (ITT) analysis virological response at Week 48 was 60% (Figure 2A).

HBsAg levels and HBV DNA levels did not change significantly, and no patient cleared HBsAg while on the waiting list for LT.

In PP analysis, median ALT level decreased from 91.0 (IQR 69.2) UI/mL at baseline to 33.5 (IQR 24.5) UI/mL at W48 (median difference 55.5, IQR = 66.75; 95% CI 24.5–109.0; *p* = 0.001).

The ALT kinetics from baseline are shown in Table 2. At W48, ALT levels normalised in 10 (66.7%) patients that achieved biochemical response. At W48, eight (53.3%) patients achieved combined, virological and biochemical responses. In ITT analysis biochemical response was reached in 50% of patients (Figure 2B).

Additionally, we did a subgroup analysis where we excluded the 3 patients that received combination therapy of BLV and PEG‐IFN. Among these 17 patients treated by BLV on monotherapy while on the waiting list 12 (70.6%) patients completed 48 weeks of BLV while on the LT list. In PP analysis, the mean HDV RNA level at baseline and at Week 48 was 5.57 log IU/mL (IQR 1.69) and 2.11 log IU/mL (IQR 2.6). The median difference HDV RNA between baseline and W48 was 3.78 log IU/mL (IQR = 3.77; 95% CI 2.13–5.65; *p* = 0.001). Virological response at Week 48 occurred in 9 (75%) of patients and non‐response in 2 (16.6%) patients. Biochemical response occurred in 7 (58.3%) patients. There was no statistically significant difference in virological or biochemical response rates between the BLV monotherapy group and the initial cohort including the 3 patients treated with combination therapy (Figure 2A,B).

Three (20%) patients lost their virological responses between Weeks 24 and 48. Two were incomplete responders, as they did not achieve a biochemical response at Week 24, and were transplanted soon after. The third patient experienced a virological relapse starting from Week 24, but he remained stable with a persistent Child‐Pugh A score during the follow‐up and maintained biochemical response (Table 3 Patient 2) Despite losing virological response he demonstrated favourable biochemical and clinical evolution by achieving biochemical response; by the end of the study 2 (10%), more patients experienced a virological breakthrough as they had consecutive increases in HDV‐RNA of ≥ 1 log_10_ IU/mL starting from 98 and 142 weeks of treatment (Table 3 patients 5 and 6 respectively).

**TABLE 3:** Baseline characteristics, treatment outcomes, and follow‐up data for decompensated HDV‐infected patients treated with bulevirtide.

*N* **°**	Age	Gender	Child baseline	Meld baseline	Ascites baseline	Ence‐phalopathy baseline	HCC baseline	Child W24	Meld W24	HCC W24	Child W48	Meld W48	HCC W48	Total treatment duration (weeks)	Virologic response achieved (week)	Virological relapse	Biochemical response achieved (week)	Liver transplantation (LT)	Adverse events related to bulevirtide	Follow‐up period (weeks)	Last status
1	67	M	C12	24	Grade 2	Grade 2	No	—	—	—	—	—	—	7	No		No	LT at Week 7 for decompensation MELD 25	No	28	Died 5 months after LT
2	35	M	C10	12	Grade 2	Grade 2	No	A6	13	No	A5	13	No	72	24	Week 48	24	Delisted	Pruritus at Week 24	72	Alive, Child A maintained biochemical response at Week 72 on treatment, no virological response
3	46	F	C11	16	Grade 3	Grade 2	No	B8	18	BCLC O	B8	16	BCLC A	48	24	No	24	LT at Week 48 for HCC BCLC A	No	95	Alive
4	42	M	C10	31	Grade 2	Grade 2	No	A5	12	No	A5	13	No	53	No		48	Delisted	No	53	Alive, biochemical response maintained at Week 53 no virological response
5	47	M	C12	32	Grade 2	Grade 2	No	B9	17	No	A5	10	No	142	48	Week 142	48	LT Week 142 for new decompensation MELD score 29	No	162	Alive
6	40	F	B7	10	Refractory	Grade 2	No	B7	9	No	A6	9	No	98	48	Week 98	48	Delisted	Fatigue	100	Alive, biochemical response maintained
7	51	M	B7	11	Grade 2	No	BCLC B	A6	9	BCLC A	A6	—	—	34	34	No	24	LT week34 for HCC BCLC A	No	126	Alive

Abbreviations: BCLC: barcelona clinic liver cancer staging system; HCC: hepatocellular carcinoma; LT: liver transplantation; MELD: model for end‐stage liver disease; *N*: number; W24: week 24; W48: week 48.

**FIGURE 3:**
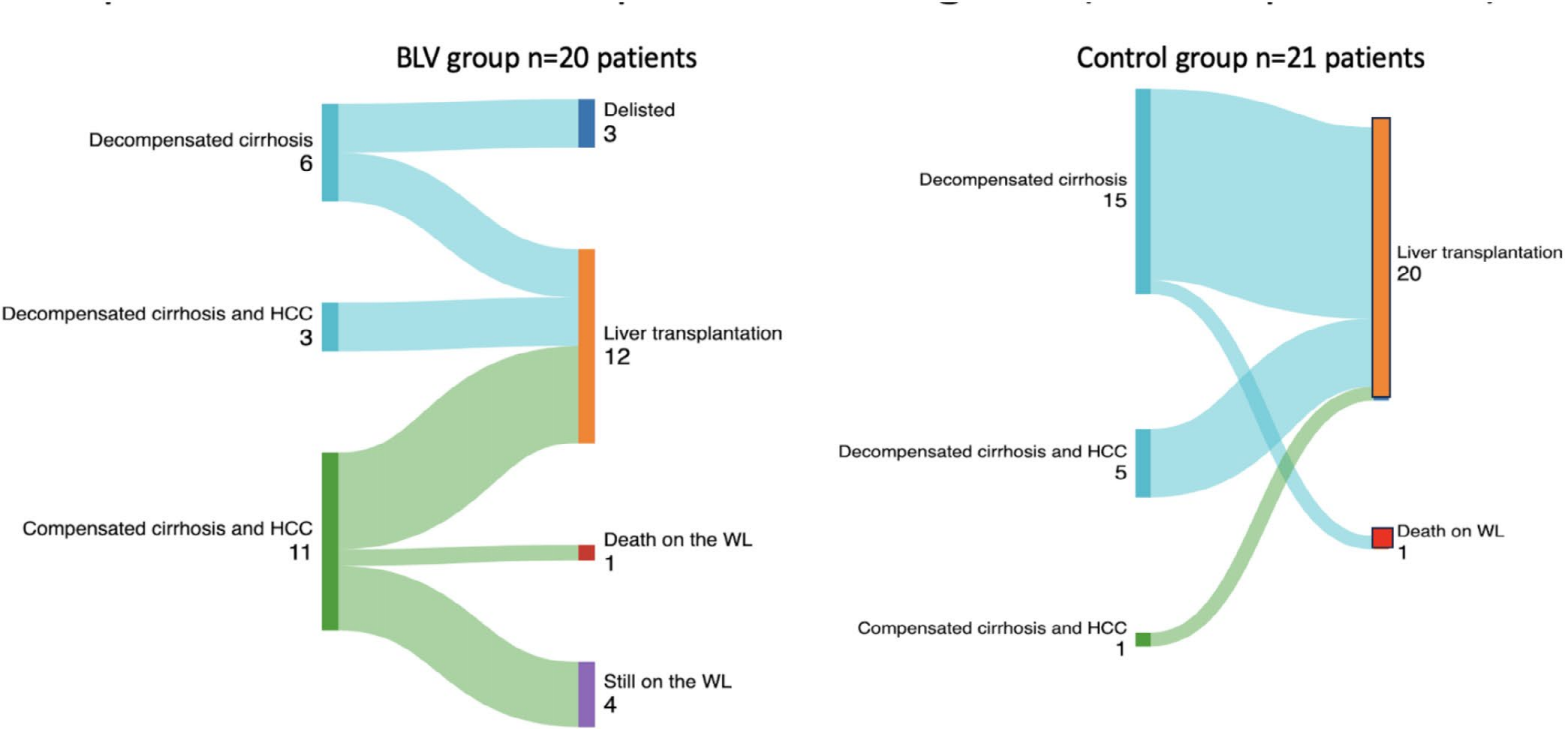
Clinical characteristics and evolution of patients once listed for liver transplant waiting list according to treatment group. BLV, bulevirtide; HCC, hepatocellular carcinoma;WL, liver transplant waiting‐list.

The clinical evolution of patients after inscription on LT is shown in Figure 3. Additionally evolution of patients after BLV initiation is detailed in Table 4 and Figure S1.

**TABLE 4:** Progression of HCC, clinical and biochemical variables for included patients (*n* = 20).

	Baseline *N* = 20	Week 24 *N* = 17	Week 48 *N* = 15	At LT *N* = 12
Active HCC	8 (40%)	10 (58.8%)	8 (53.33)	8 (66.6%)
BCLC stage O	4 (50%)	2 (20%)	2 (25%)	0
BCLC stage A	1 (12.5%)	6 (60%)	4 (50%)	8 (100%)
BCLC stage B	3 (37.5%)	2 (20%)	1 (12.5%)	0
BCLC stage C	0	0	1 (12.5%)	0
LSM KPa^a^	24.16 (10–58)	—	16 (11.5–16.2)	
Child‐Pugh score				
A (5–6 points)	13 (65%)	11 (64.7%)	12 (80%)	6 (50%)
B (7 points)	2 (10%)	3 (17.6%)	2 (13.33%)	2 (16.66%)
C (10–15 points)	5 (25%)	3 (17.6%)	1 (6.7%)	4 (33.34%)
MELD score	9 (6–32)	8 (6–18)	9 (6–16)	12 (6–31)
ETV or TDF treatment	18 (90%)	16 (94.1%)	14 (93.3%)	12 (100%)
Ongoing PegIFNα treatment^b^	3 (15%)	0	0	0
Laboratory tests				
Platelet count × 10^3^/mmol/L	94 (50.2–285.5)	92 (23–153)	104 (25–138)	70 (28–246)
AST, U/L	78 (65.5–125)	60 (25–477)	52 (20–70)	79.5 (26–322)
ALT, U/L	91 (60–135.5)	42 (14–512)	33.5 (8–93)	62 (21–335)
Bilirubin, mmol/L	20.7 (10–444)	18 (6–182)	13.5 (9–29)	18.5 (7–525)
Prothrombin time %	78 (26–100)	75 (39–100)	72 (39–100)	59.5 (27–100)
Biochemical response (ALT normalisation)	—	6 (35.29%)	10 (66.6%)	3 (25%)

*Note:* Data shown as medians.

Abbreviations: ALT, alanine aminotransferase; AST, aspartate aminotransferase; BCLC, barcelona clinic liver cancer; ETV, entecavir; HBV, hepatitis B virus; HCC, hepatocellular carcinoma; LSM, liver stiffness measurement; MELD, model for end‐stage liver disease; PegIFNα, PEGylated interferon alpha; TDF, tenofovir disoproxil fumarate.

^a^ *n* = 9 patients.

^b^ *n* = 2 patients received 180 g/week and 130 μg/week.

During the study, MELD scores increased in two patients, rising from 11 at baseline to 25 at LT, respectively from 9 at baseline to 31 at LT. The first patient received less than 24 weeks of bulevirtide treatment and had a Child‐Pugh score of C10 at LT, and the second had compensated Child‐Pugh A5 cirrhosis at baseline and presented with variceal bleeding at W50.

**FIGURE 4:**
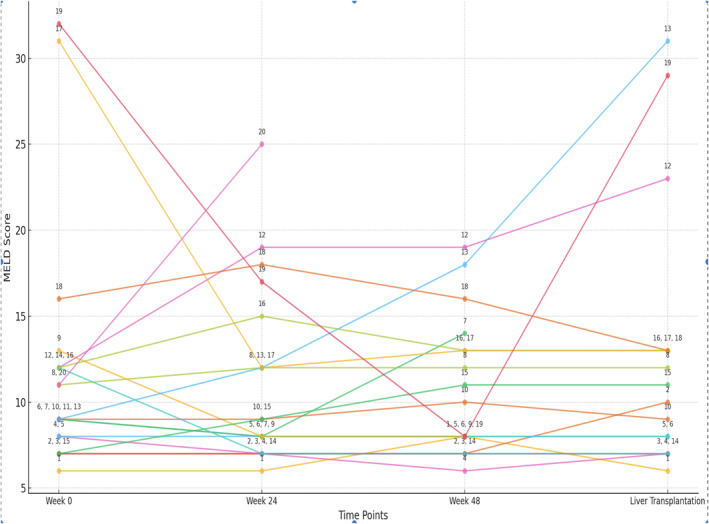
MELD score evolution during bulevirtide treatment for *N* = 20 patients (each number represents a different patient).

Overall MELD evolution did not show significant MELD changes during the treatment course, with an overall median MELD of 9 (±6.13). The mean change for all patients during BLV study was +1.53 points. A total of 5 (25%) patients demonstrated MELD reduction below 15. The evolution of MELD scores during the study period is shown in Figure 4.

### 3.3 Virological and Biochemical Responses in Bulevirtide‐Treated Child C Patients

Among the five (25%) decompensated Child‐Pugh C patients treated with bulevirtide (identified by patient numbers in Table 3), virological response at week 48 was achieved in two patients (patients 3 and 5).

Patient 1 underwent early liver transplantation at Week 7 after bulevirtide initiation because of worsening of liver function, while patient 2 initially achieved a virological response at Week 24 but experienced a virological relapse by Week 48 of treatment.

Patient 4 did not demonstrate a virological response throughout the study period but showed progressive improvement in liver function with a MELD score that decreased from 31 to 13 after 48 weeks of bulevirtide treatment. The patient's liver function improved by Week 24, with ALT levels decreasing significantly from 10‐times the upper limit of normal (ULN) at baseline to twice the ULN and normalised by Week 48. Viral load decreased at Weeks 24 and 48 but less than 1 log decrease, so it was considered a virological non‐responder. The patient could be delisted from the LT waiting list and antiviral treatment was the only contributing factor.

With respect to biochemical response in Child C patients, two patients (patients 2 and 3) achieved a response by Week 24, and two additional patients (patients 4 and 5) demonstrated a biochemical response at Week 48. Patient 5 showed continued improvement in liver function, with a Child‐Pugh score improving from B9 at Week 24 to A6 at Week 48, coinciding with both biochemical and virological responses. The patient was not removed from the transplant list but had a temporary contraindication due to improvement in liver function. However, he was ultimately transplanted at Week 142 following further decompensation unrelated to BLV use, but rather to disease progression. Detailed data are presented in Table 3.

### 3.4 Virological and Biochemical Responses in Bulevirtide Treated Child B Patients

At the initiation of BLV, one patient (Table 3, patient 6) presented with refractory ascites. Cirrhosis was classified as Child‐Pugh B7 or MELD 10 and had grade 2 encephalopathy. The patient was listed for LT because a transjugular intrahepatic portosystemic shunt (TIPS) was contraindicated. By Week 24, the MELD score had slightly decreased to 9, while the Child‐Pugh score remained B7. By Week 48, both virological and biochemical responses were achieved, resulting in an improvement in the Child‐Pugh score to A6 and a further reduction in the MELD score to 8. The ascites and encephalopathy had fully resolved by Week 48, and the patient showed significant clinical improvement over the 33‐month follow‐up period, ultimately leading to its removal from the liver transplant waiting list.

Moreover, one patient (12.5%) with hepatocellular carcinoma (HCC) at baseline had cirrhosis classified as Child‐Pugh B7 and HCC BCLC stage B (Table 3, patient 7). Chemoembolization was initially contraindicated due to liver function impairment, and the patient was listed for LT. Bulevirtide therapy was initiated, and by Week 24, a biochemical response was observed, with an improvement in liver function to Child‐Pugh A6. This improvement allowed the patient to become eligible for chemoembolization and downstaging therapy, which was previously unfeasible. At the time of LT, the patient was successfully downstaged to BCLC stage A, and exhibited a combined virological and biochemical response.

In summary, among the 7 decompensated patients with Child‐Pugh B or C cirrhosis, 3 (42.9%) patients were delisted from the transplant waiting list, including 2 (40%) Child C patients and 1 (50%) Child B patients. In terms of virological response, 3 (42.8%) patients achieved a response at Week 48, including 2 (40%) Child C patients and 1 (50%) Child B patients. Biochemical response at Week 48 was observed in 5 (71.4%) patients, including 4 (80%) Child C patients and 1 (50%) Child B patients. Liver function improvement was noted in 6 (85.7%) patients, including 100% of Child B patients and 4 (80%) of Child C patients.

### 3.5 Virological and Biochemical Evolution in Bulevirtide Treated HCC Patients

Seven (35%) patients had active HCC and compensated CHILD A cirrhosis at baseline and were treated with BLV while on the waiting list and having downstaging treatment. Five (25%) patients with compensated Child‐Pugh A cirrhosis developed new HCC at BCLC A stage while on BLV treatment. Specifically, two patients developed HCC at Week 24, one at Week 48, one at Week 120, and another at Week 92 of treatment, which was the reason for listing them for LT. The patient who developed HCC at Week 92 had prior radiofrequency ablation but was not prioritised for LT, as the HCC was inactive and French guidelines prioritise only upon reactivation. By the end of the study, three of these patients underwent LT, after a mean waiting time of 50.7 weeks (range 38.2–63.1 weeks). The data are presented in Table 1 and Figure 2. The development of HCC did not appear to be related to bulevirtide treatment but rather to disease progression.

### 3.6 Liver Transplantation for HDV‐Infected Patients

Among the 20 patients in the BLV group, 12 (60%) patients underwent LT: one (8.3%) after less than 24 weeks of BLV, two (16.6%) after 24 weeks of BLV, and nine (75%) after at least 48 weeks of BLV. The median time from BLV treatment start to LT was 49.97 (± 37.39) weeks. For patients receiving a LT, the median log IU/mL HDV RNA at baseline and at LT were 5.43 (±1.91) and 3.11 (±2.44), respectively, and there was a significant decrease in HDV RNA between baseline and the LT (*p* = 0.006). Among the eight patients with active HCC at baseline, five (62.5%) underwent LT, all with BCLC A stage at LT after a mean waiting time of 32.57 weeks (range 30.2–82.4).

Among the 21 patients in the control‐group, a total of 20 patients (95.2%) underwent LT after a mean waiting time of 17.2 weeks (range, 1–32 weeks) following their listing for transplantation. One (4.8%) patient died while on the waiting list. Reasons for BLV treatment abstention were decompensated disease at inscription on the LT waiting list and need for off‐label prescriptions of bulevirtide. At 3 months, transplant free survival was 36.7% (95% CI: 16.9–56.8) months in the control group versus 76.9% (95% CI: 44.2–91.9) in the BLV group (*p* = 0.00714). In a multivariate Cox regression analysis that included BLV treatment, age, and MELD score, only baseline MELD score was predictive of transplant‐free survival (HR 1.11, 95% CI: 1.04–1.18, *p* < 0.001).

### 3.7 Follow‐Up After LT

After LT, the median follow‐up was 18.76 (± 10.9) months. Within this period, two patients (16.7%) died in the BLV group: one at 11 months due to de novo cholangiocarcinoma and another at 5.5 months from an unknown cause. These deaths were not related to the BLV treatment. None of the patients in the control group died. Post‐transplant HDV RNA was undetectable in all patients, and HBV DNA and HBs antigen were also negative. Post‐transplant antiviral treatment consisted of hepatitis B immunoglobulin (HBIg) administration, with 93.75% of patients receiving subcutaneous doses of 500 IU weekly and 3.12% receiving intravenous doses of 6000 IU monthly, as long‐term prophylaxis. Additionally, NAs were prescribed, with 53.1% of patients on entecavir and 43.6% on tenofovir and 3.1% on lamivudine.

### 3.8 Treatment Tolerance and Safety

Among the 17 patients receiving at least 24 weeks of bulevirtide, two (11.7%) developed injection site reactions and pruritus and two (11.7%) reported fatigue and headache. After 24 weeks of bulevirtide, one (12.5%) patient developed refractory ascites, which was attributed to liver disease evolution rather than to bulevirtide.

Among the 15 patients who completed 48 weeks of treatment, at W48, one (6.6%) had moderate fatigue and another (6.6%) had pruritus. No worsening of liver function was directly imputable to BLV. One Child‐Pugh class A patient with HCC presented with a transaminase flare at W24 attributed to chemoembolization rather than BLV, which rapidly resolved and did not impact BLV treatment. No ALT flares or significant ALT elevations with BLV utilisation were observed.

Among the five Child‐Pugh C patients, treatment tolerance was good. One patient experienced pruritus, and another reported fatigue during the treatment period, but deterioration in liver function was not related to BLV treatment.

## 4 Discussion

Chronic hepatitis delta (CHD) remains a significant global health concern, as patients are at high risk of developing liver cirrhosis, decompensated end‐stage liver disease, and HCC. Degasperi et al. [19] provided the first report on the efficacy and safety of 2 mg bulevirtide monotherapy for 48 weeks in patients with compensated cirrhosis and clinically significant portal hypertension with or without active HCC. In their study, liver function improved in four out of five patients with Child‐Pugh A6 disease, which represents the most important clinical parameter [12]. Recently, Dietz‐Fricke et al. [20] reported real‐world data on the off‐label use of bulevirtide in 19 patients with decompensated Child‐Pugh B liver disease, revealing similar safety and efficacy: their study demonstrated a virologic response rate of 74% after an average treatment duration of 17 weeks, and 47% of patients experienced a clinical improvement in liver function, mainly due to the resolution of ascites. It is unknown whether treating patients with advanced liver disease with bulevirtide could be an alternative to LT or prompt delisting due to clinical improvement. Our multicenter study provides supplementary real‐world data on the efficacy and safety of bulevirtide in CHD patients with advanced liver disease who are undergoing evaluation for LT or are waiting for LT. Our results demonstrate that 48 weeks of treatment with bulevirtide is well tolerated and associated with significant virological (73.3%) and biochemical responses (66.6%) in the majority of these patients. Notably, this included patients with decompensated Child‐Pugh C cirrhosis, where 60% (3 out of 5) improved to Child‐Pugh A status, and three (15%) patients from the total cohort were subsequently delisted due to liver function improvement. These safety and efficacy profiles have also been applied to patients with HCC undergoing downstaging therapy while on LT waiting list.

Our findings are consistent with other real‐world studies on compensated or decompensated Child‐Pugh B cirrhosis where bulevirtide use demonstrates antiviral efficacy with respect to HDV RNA levels [19, 20, 21]. The adverse events observed in our study, such as injection site reactions, fatigue and headache, were generally mild and manageable, confirming the reported favourable safety profile [19, 20].

As this was a real‐life, multicentric study, the decision to treat patients with BLV monotherapy or combination therapy (PEG‐IFN and BLV) was left to the discretion of the investigators. In our study three patients had combination therapy for less than 24 weeks and all of them had compensated cirrhosis prior to PEG‐IFN initiation. Adding PEG‐IFN did not change outcomes in term of virological or biochemical response, but the number of treated patients was small (15%) and treatment duration off with less than 24 weeks.

The rationale for initiating PEG‐IFN combination therapy comes from findings from our French BuleDelta study (NCT04166266), which demonstrated that 72.2% of patients receiving combination therapy achieved a virological response after 96 weeks, compared to 55% in the monotherapy group (*p* < 0.01). Furthermore, the same study reported that statistically significant virological and biochemical changes between the combination therapy group and the monotherapy group were seen only after 48 weeks of treatment (*p* < 0.0001) [22].

Additionally, we performed a comparative analysis with an untreated similar patient cohort from all the participating centers. This analysis showed that patients treated with bulevirtide presented with less severe liver disease at the time of listing, as evidenced by lower Child‐Pugh and MELD scores, compared with untreated patients, and exhibited a higher prevalence of HCC. We highlight the case of a Child‐Pugh B patient for whom bulevirtide treatment led to significant improvement in liver function, allowing for successful HCC downstaging and prevention from transplant waitlist dropout. Moreover, biochemical response to bulevirtide allowed five (62.5%) HCC patients from our cohort to receive loco‐regional therapy while on the waiting list, underscoring the potential of bulevirtide to enhance access to HCC treatment options, enabling tumour downstaging and reducing dropout rates.

In univariable analysis three‐month transplant‐free survival rate was significantly higher in the bulevirtide‐treated group (76.9%, 95% CI: 44.2–91.9) compared to the untreated group (36.7%, 95% CI: 16.9–56.8; 0.007), suggesting benefit from bulevirtide treatment in improving short‐term outcomes in patients on the waiting‐list. However, due to the small size cohort and differences in clinical characteristics between treated and untreated patients, in multivariable analysis, only MELD predicted of transplant‐free survival (HR, 1.11; 95% CI: 1.04–1.18, *p* < 0.001).

To our knowledge, this is the first study to report safe off‐label use of bulevirtide in a cohort of decompensated Child C patients and reports delisting of 15% of patients following significant liver function improvement induced by bulevirtide. This outcome was not observed in any patient from the untreated cohort, where neither Child‐Pugh score nor MELD score significantly differed between the time of listing and LT. Interestingly, one patient from the treated cohort experienced liver function improvement despite not achieving a virological response, which is consistent with prior studies suggesting that biochemical response alone can lead to clinical benefit [21].

Based on our experience, bulevirtide does not seem to alter the evolution of decompensated liver disease, as also suggested by Diet‐Fricke et al. [20], but may contribute to disease stabilisation in some patients. Biochemical response at week 48 was associated with liver function improvement in 85% of cases while persistent abnormal liver function was linked to poor prognosis. However, the small sample size limits the ability to draw statistically significant conclusions We observed that the MELD score did not change significantly from baseline to LT, possibly because of the small sample size limiting statistical power; therefore, we could not determine a specific MELD score that predict the futility of bulevirtide treatment consecutively we cannot recommend a definitive clinical approach. However, our findings suggest that in clinical practice bulevirtide may benefit and be proposed to patients with a lower MELD score (< 20) or those needing MELD exceptions, such as those with refractory ascites or untreatable HCC with AFP score ≤ 2 and MELD < 20, who face delays in accessing LT. It is likely futile to treat patients with MELD scores > 25, as they have a low chance of rapid improvement and may benefit more from transplantation. Since transplant patients are already protected from HBV and HDV recurrence by treatments such as NA and HBIg, further reduction of HDV RNA in patients undergoing LT is not needed from a strictly virological point of view. However, in the context of organ shortages, it is important to develop a strategy to optimise transplant utility and avoid futile transplantation.

We also reported a 10% rate of late virological breakthrough. Although we did not perform a sequencing analysis, it is possible that these patients had NTCP polymorphisms rather than treatment‐induced resistance, as suggested by Hollnberger et al. [23] and they may benefit in the near future from other emerging therapies [24]. Another hypothesis for incomplete viral response or virological breakthrough could be related to possible differences in BLV kinetics and exposure between patients with decompensated liver disease and those with preserved liver function, potentially reducing drug efficacy but this needs further research.

This study has several limitations. Its retrospective design and small sample size may restrict the generalizability of the findings. Treatment decisions and timing of treatment introduction was at the investigators' discretion, moreover treatment was prescribed off‐label in cases of decompensated cirrhosis. Additionally, patients treated with bulevirtide had a better initial liver function which may have influenced outcomes. Also, the study was conducted in France where treatment is reimbursed and included patients from tertiary referral centers. Consequently, larger, prospective controlled trials with extended follow‐up periods are necessary to validate these results. Despite these limitations, our study reports the outcomes and safety of bulevirtide treatment in the setting of LT. We also demonstrated favourable treatment outcomes in decompensated CHILD C cirrhosis that resulted in delisting of patients while in HCC patients treatment favoured downstaging strategies. In addition, we performed a comparative analysis with a similar untreated cohort.

In conclusion, our study highlights the potential use of bulevirtide in patients with CHD on the LT waiting list. Bulevirtide demonstrated a favourable efficacy and safety profile along with notable clinical benefits. If confirmed in larger studies, these findings suggest that bulevirtide could be effective in improving liver function in patients with decompensated cirrhosis and may facilitate bridging therapies to transplantation, particularly for those with hepatocellular carcinoma. Therefore, Bulevirtide could be a game‐changer in pretransplant settings.

## Author Contributions

All authors contributed to the data interpretation and reviewed, revised, and approved the manuscript. Drs. Meszaros and Pageaux had full access to all the data in the study and took responsibility for the integrity and accuracy of the data analysis. Concept and design: Drs Meszaros, Pageaux, Dumortier, Dharancy. Statistical analysis: Dr. Meszaros.

## Ethics Statement

The ethics committee of the University Hospital of Montpellier granted ethical approval.

## Conflicts of Interest

The authors declare no conflicts of interest.

## Supporting information


Figure S1.

## Data Availability

The data that support the findings of this study are available on request from the corresponding author. The data are not publicly available due to privacy or ethical restrictions.
