# Supplementary material for: Bulevirtide in Chronic Hepatitis D Patients Awaiting Liver Transplantation Results From a French Multicentric Retrospective Study
Source: Liver Int. 2025 Feb 17;45(3):e70033. doi: 10.1111/liv.70033 (PMC11831879; doi:10.1111/liv.70033)
Supplement: Supplementary file 1 — Figure S1. [file LIV-45-0-s001.docx]

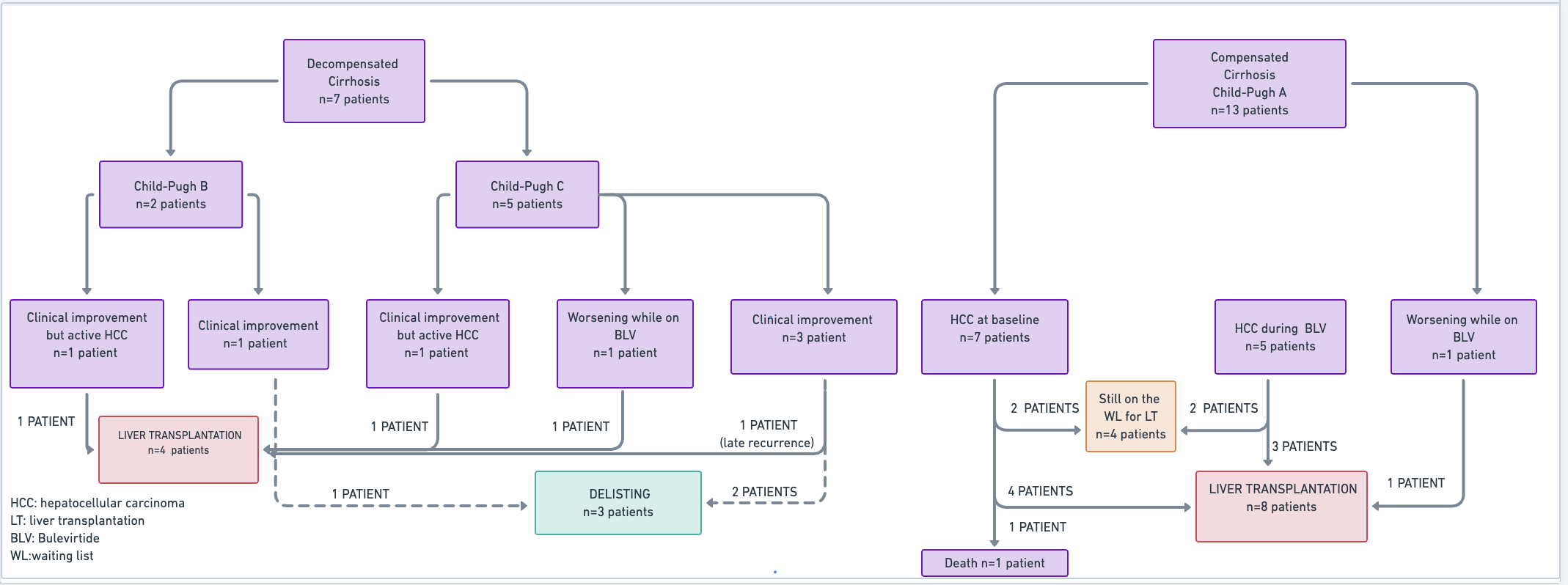


**Supplementary Figure 1:** Clinical evolution of patients in bulevirtide group after bulevirtide initiation
